# Efficacy of a facial-aging web app on sun protection behaviors among primary school students in Iran: a randomized controlled trial

**DOI:** 10.1186/s12889-024-18241-2

**Published:** 2024-03-07

**Authors:** Hassan Okati-Aliabad, Esmat-Sadat Hosseini, Mohammad Ali Morowati Sharifabad, Mahdi Mohammadi, Mohamad Ebrahimzadeh Ardakani, Amir Hossein Talebrouhi

**Affiliations:** 1 Health Promotion Research Center, Zahedan University of Medical Sciences, Zahedan, Iran; 2 Health Education and Health Promotion, Shahid Sadoughi University of Medical Sciences, Yazd, Iran; 3 Department of Health Education & Promotion, Shahid Sadoughi University of Medical Sciences, Yazd, Iran; 4 Department of Dermatology, Shahid Sadoughi University of Medical Sciences, Yazd, Iran; 5 Department of Computer Engineering, Imam Reza International University, Mashhad, Iran

**Keywords:** Ultraviolet (UV), Sun protection, Student, Iran

## Abstract

**Background:**

Skin cancers resulting from excessive exposure to solar ultraviolet (UV) radiation are on the rise. This study aims to investigate the impact of facial-aging app intervention on promoting safe and healthy behaviors and its influence on reducing students' UV exposure.

**Method:**

Utilizing a Pretest–Posttest repeated-measures design, we developed a theory-guided web app on the WhatsApp platform, named the Sunshine and Skin Health app. This app allows users to visualize their altered faces in three stages of adolescence, middle age, and old age based on sun protection behavior. The intervention continued within WhatsApp, incorporating 27 health messages grounded in the PMT theory, eight educational files, and a skin cancer video clip. The primary outcome is the change in sun protection behavior between the two groups (intervention and control) immediately after the intervention (T2) and the secondary outcome is the change in sun protection behavior between the two groups at 3 months follow-up (T3). The data are analyzed in SPSS 22 and a significance level of 0.05 is considered.

**Results:**

The results revealed no significant difference between the two groups before the intervention. However, in the intervention group, there were significant differences in the utilization of sunglasses, hats, and sunscreen in the last month, as well as sunscreen reapplication after washing their hands and face, both immediately after the intervention and at the 3-month follow-up, compared to the control group (*P* = 0.001). Furthermore, a significant intervention effect, time effect, and interaction effect between group and time were observed in behaviors related to using sunscreen in the last month and sunscreen reapplication after washing hands and face (*P* = 0.001). Specifically, the intervention group exhibited a significant difference from Time 1 to 2 and from Time 1 to 3 (*p* = 0.001), but no significant difference from Time 2 to 3. In contrast, the control group did not show any significant differences over time.

**Conclusions:**

This study indicated that the Facial-Aging web app can effectively encourage safe behaviors in sunlight. To ensure the maintenance and sustainability of these behaviors over the long term, it is crucial to consider implementing booster sessions.

**Trial registration:**

Iranian Registry of Clinical Trials IRCT20200924048825N1. Registered prospectively on 8 February 2021.

**Supplementary Information:**

The online version contains supplementary material available at 10.1186/s12889-024-18241-2.

## Background

Solar radiation is classified as a carcinogen and serves as the primary source of human exposure to UV radiation [1]. With nearly 90% of the global population residing in areas where the annual peak UV index (UVI) exceeds 10, the potential for UV radiation exposure is significantly heightened. However, the actual individual dose absorbed is contingent on behavior [2]. UV light poses a major health concern, contributing to DNA damage and immunosuppression, which mediate skin cancer carcinogenesis. Additionally, UV exposure is implicated in the development of conditions such as cataracts, pterygium, and potentially age-related macular degeneration [3, 4].

On the flip side, sun exposure offers numerous health benefits; it plays a crucial role in vitamin D synthesis, which is essential for absorbing calcium, promoting bone growth, and supporting various bodily functions [5]. The annual UV index average map in Iran indicates a significantly high index in the country's southern half. The highest levels were observed in the southern provinces, particularly in Sistan and Balochistan, highlighting the increased risk of eye damage and susceptibility to skin diseases among the population [6].

Childhood and adolescence represent critical periods for learning and adopting sun-protection behaviors [7]. Preventing skin cancer in children is of utmost importance, as their susceptibility to skin cancer in adulthood increases with greater exposure to ultraviolet (UV) radiation during childhood. Consequently, sun protection practices acquired in the early years tend to evolve into lifelong habits [8]. The primary foundation for preventing skin cancer lies in effectively managing UV exposure to reduce or prevent chronic sun damage, particularly skin aging and skin cancer [9]. To mitigate damage caused by sunlight, adopting the following behaviors is recommended: seeking shade, using appropriate sunscreen (including a wide-brimmed hat, matte UV sunglasses, and long-sleeve shirts), minimizing exposure or avoiding sunlight during peak hours (10 am to 4 pm), applying sunscreen with SPF 30 or higher in uncovered skin areas, and steering clear of artificial sources of UV rays, such as fluorescent lamps [10, 11].

The Protection Motivation Theory, introduced by Rogers in 1975, is frequently employed to elucidate how individuals adopt protective measures against various diseases. PMT, a social cognition theory, encompasses the structures of perceived susceptibility, perceived severity, fear, reward, response efficacy, self-efficacy, and protection motivation [12].

### Aims of the study

This study, grounded in the Protection Motivation Theory (PMT), aims to investigate the effectiveness of a Facial-Aging web app intervention among primary school students. Using a longitudinal intervention design with assessments at three points over three months, we conducted two comparisons:

1. TEST 1–TEST 2: Is the intervention effective?
2. TEST 1–TEST 3: Are intervention effects sustained?

- Hypothesis 1: The sun protection behaviors immediately following the intervention (T2) will differ between the two groups.
- Hypothesis 2: At follow-up (Time 3), participants in the intervention group will exhibit a superior pattern of sun-protective behaviors compared to the pre-intervention (pre-test) phase.

## Methods

### Participants and procedure

**Fig. 1:**
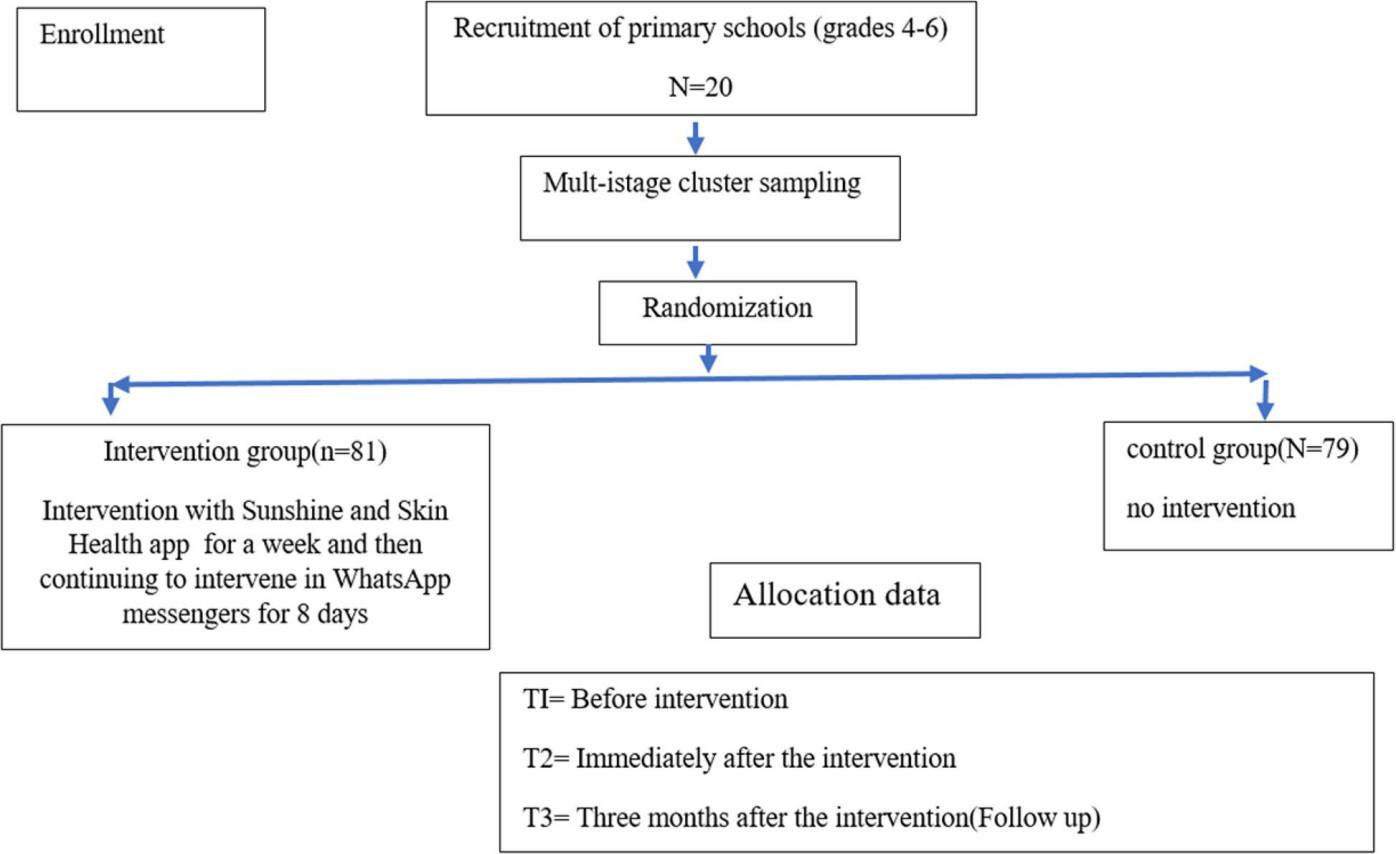
Study diagram

This study was conducted from September 2021 to July 2022, encompassing participant recruitment, data collection, and intervention. Employing a pretest–posttest repeated measures design with two parallel groups, the research focused on 10- to 12-year-old elementary school students (grades 4–6) and spanned three assessment points over three months. The sampling process utilized a multi-stage cluster random approach. A Facial-Aging web app intervention was implemented randomly among half of the sample, while the other half served as the control group. The study protocol received approval from the Ethics Committee of Shahid Sadoughi University of Medical Sciences (approval numbers: IR.SSU.SPH.REC.1399.135). Data collection occurred online, utilizing the messaging platform WhatsApp (see Fig. 1).

### Randomization

Zahedan was stratified into 5 districts based on socioeconomic status. Within each district, one girls' school and one boys' school were randomly selected for the intervention group. To mitigate information dissemination, a school with the greatest distance in each district was selected for the control group. Consequently, 5 schools were allocated to the intervention group, and 5 schools were assigned to the control group. In each school, students from the 4th, 5th, and 6th grades were randomly selected in proportion to the school's size. A total of 160 students from grades 4 to 6 were recruited and divided into an intervention group (*n* = 81) and a control group (*n* = 79).

All male and female students enrolled in the 4th to 6th grades of both public and private schools, possessing access to the Internet, social networks (WhatsApp), mobile phones, tablets, and laptops, were eligible to participate in the study. Students expressing unwillingness to cooperate during the research project and data collection, as well as those who did not complete the pre-test or post-test questionnaire, were excluded from the study.

### Intervention

To implement the intervention, the free messaging platform WhatsApp was utilized both for intervention delivery and as one of the data collection tools. Students were randomly assigned to intervention and control groups, each having a dedicated WhatsApp group (Sunshine and Skin Health Intervention Group and Sunshine and Skin Health Control Group). The study goals were thoroughly explained to both groups. Considering the absence of intervention in the control group, ethical considerations were maintained by implementing interventions for the control group post-study completion.The initial session for the Sunshine and Skin Health app took place on a holiday (April 2022, Friday). Students had the option to call via telephone or engage in WhatsApp chats with the researcher whenever needed. They were given a week to practice using the Sunshine and Skin Health app. Following app usage, a one-week invitation message was displayed, and the intervention continued on WhatsApp. The questionnaire was completed immediately after the intervention (T2) and again after a 3-month follow-up (T3) within the program. Written consent from students and parents (electronically) was obtained before questionnaire completion and recorded in the experimental database.

### Sunshine and skin health app intervention

**Fig. 2:**
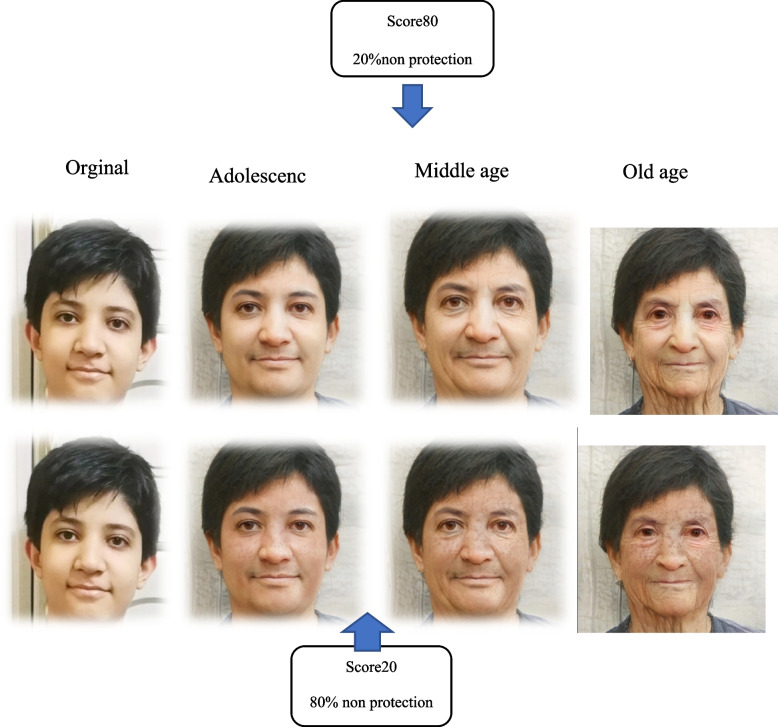
Program implementation graphics in adolescence, middle age, and old age phases

The Sunshine and Skin Health app is a stand-alone, web-based application designed to deliver an educational intervention on skin cancer prevention through sun protection behaviors. This innovative app incorporates artificial intelligence technology. In the initial phase, users access the app by following a provided link and, upon uploading a photo, the app utilizes artificial intelligence to determine skin color, eye color, gender, and age. In the subsequent step, users respond to behavioral questions and witness the transformation of their faces in three stages: adolescence, middle age, and old age. The third step involves the app delivering educational messages and recommendations tailored to each individual's skin color and eye color (see Fig. 2).

### WhatsApp messenger app intervention

The intervention persisted on WhatsApp. Following the completion of interaction with the Sunshine and Skin Health app, a one-week invitation message was presented. This intervention comprised 27 health messages grounded in PMT theory, along with eight educational files and a skin cancer video clip. These materials were distributed via WhatsApp over one week, from 10 am to 4 pm. Each day concluded with a question related to health messages, educational files, or video clips, prompting participants to respond with their answers via voice or text. The messages draw upon scientific literature covering topics related to enhancing sun protection behavior [11, 13]. Following an extensive literature review on sun protection interventions [14–20], the educational content was crafted, encompassing guidance on using sunscreen, seeking shade, wearing a brimmed hat and sunglasses, and adopting long-sleeved clothing. This intervention spanned eight days on the free WhatsApp platform. The delivery of health messages, grounded in PMT as the theoretical framework of this study, followed a regular schedule as outlined in Table 1.

**Table 1:** Schedule of health messages in the WhatsApp Messenger App

Days of the Week	Time	PMT constructs	Messages Number
Saturday	10 am to 4 pm	Perceived Susceptibility and Perceived Severity	4
Sunday	10 am to 4 pm	Fear	2
Monday	10 am to 4 pm	Self-Efficacy, Response Cost, and Perceived Response Efficacy	4
Tuesday	10 am to 4 pm	Self-Efficacy, Response Cost, and Perceived Response Efficacy	4
Wednesday	10 am to 4 pm	Self-Efficacy, Response Cost, and Perceived Response Efficacy	4
Thursday	10 am to 4 pm	Reward	3
Friday	10 am to 4 pm	Protection Motivation	3
Saturday	10 am to 4 pm	Protection Motivation	3

## Measures

### Demographic survey

Socio-demographic characteristics, including age, gender, grade, parent's education level, and parents' job, were collected through a survey completed by the students.

#### Sun protection behaviors scale

Students self-reported their sun protection behaviors using a 7-item scale. Participants rated each item on a 5-point Likert scale ranging from 1 to 5 (never to always) including:

1. How often did you use sunscreen last month?
2. When exposed to intense sun during outdoor activities (e.g., fun, games, shopping), how often do you seek shade?
3. How often do you wear a wide-brimmed sun hat when exposed to sunlight during peak hours (10 am to 4 pm)?
4. How often do you wear long-sleeved clothing when exposed to harsh sun?
5. How often do you wear sunglasses when exposed to sunlight during peak hours (10 am to 4 pm)?
6. How often do you use sunscreen when exposed to sunlight during peak hours (10 am to 4 pm)?
7. Do you use sunscreen again after washing your hands and face?

The validity of the questionnaire items was assessed through face validity and qualitative content validity. A panel of 10 experts in health education and promotion, and dermatology examined the items, providing feedback on simplicity, clarity, readability, grammar, wording, scoring, and relevance. Adjustments were made based on their input, addressing unclear questions and minor wording errors. Reliability was evaluated using Cronbach’s α. Thirty students completed the questionnaire, resulting in a Cronbach’s α of 0.66.

### Analytical procedure

Descriptive statistics, including mean (standard deviation, SD) for quantitative variables and frequency (percentage) for qualitative variables, were employed to characterize the data. The Chi-square test was applied to compare demographic variables between the intervention and control groups. To assess behavior differences before the intervention, an independent samples T-test was conducted with Bonferroni correction. Repeated Measures ANOVA was employed to examine behavior variations between the two groups across the three-time points. Additionally, an independent samples T-test was utilized to compare behavior between groups at each time point. All statistical analyses were performed using SPSS 22, with a significance level set at less than 0.05.

## Results

### Sample characteristics

Demographic characteristics are presented in Table 1. The sample consisted of 160 students divided into two groups: intervention (57 girls [70.4%] and 24 boys [29.6%]) and control (63 girls [79.7%] and 16 boys [20.3%]). The mean [SD] age was 10.88 [1.8] years. Notably, relevant characteristics were well-balanced between the intervention and control groups. A significant portion of participants in both groups attended the 5th grade (intervention n = 31, 38.3%; control *n* = 33, 41.8%). Regarding parental education, 39.5% of fathers and 38.8% of mothers had an academic education. Employment status varied, with 45.7% of fathers employed, and the majority of mothers (75.0%) identified as housewives. No statistically significant differences were found between the control and intervention groups (Table 2).

**Table 2:** Sample demographic characteristics

Variable		Intervention Group (*n* = 81)		Control Group (*n* = 79)		*P* Value
		Number	Percent	Number	Percent	
Gender	Boys	24	29.6	16	20.3	0.171
	Girls	57	70.4	63	79.7	
Grade	Grade 4	24	29.6%	28	35.4%	0.407
	Grade 5	31	38.3%	33	41.8%	
	Grade 6	26	32.1%	18	22.8%	
Mother's Education	Illiterate	4	4.9%	7	8.9%	0.525
	Primary school	4	4.9%	4	5.1%	
	middle school&High school	12	14.8%	17	21.5%	
	Diploma	30	37.0%	29	36.7%	
	Academic	31	38.8%	22	27.8%	
Father's Education	Illiterate	7	8. 6%	3	3.08%	0.462
	Primary school&middle school&High school	11	13.6%	12	15.2	
	Diploma	31	38.3%	37	46.8%	
	Academic	32	39.5%	27	34.2%	
Mother's Job	Housewife	60	75.0%	49	61.2%	0.148
	Employed	20	25.0%	31	38.8%	
Father's Job	Employed	37	45.7%	36	45.6%	0.093
	Worker	11	13.6%	11	13.9%	
	Self-employed	33	40.7%	32	40.5%	

### Intervention effects

Before the intervention (Time 1), no significant difference in sunscreen use in the last month was found between the two groups (*P* = 0.983). However, a significant intervention effect (*P* = 0.001), time effect (*P* = 0.001), and interaction effect between group and time (*P* = 0.001) were observed. Sun protection behavior significantly differed between the intervention and control groups immediately after the intervention (*P* = 0.001) and after the 3-month follow-up (*P* = 0.001). Specifically, in the intervention group, a significant difference was observed from Time 1 to 2 (*P* = 0.001), Time 1 to 3 (*P* = 0.001), and Time 2 and 3 (*P* = 0.001). Conversely, no significant difference over time was noted in the control group (Table 3).

**Table 3:** Sun protection behavior

Behavior	Groups	Before Intervention T1	Immediately after intervention T2	Three months After the intervention T3	Repeated Measure ANOVA (*p*-value)				Repeated Measure ANOVA (*p*-value)			Independent samples T-test		
		Mean ± SD	Mean ± SD	Mean ± SD	Effects	F	P	ɳ2	Time	Groups		Intervention& Control		
										Control	Intervention	T1	T2	T3
Use of Sunscreen (Last Month)	Control Intervention	2.69 ± 1.53	2.78 ± 1.18	2.49 ± 1.38	Time	11.521	0.001	0.068	T1&T2	1.000	0.001	*P* = 0.983	*P* = 0.001	*P* = 0.001
		2.69 ± 1.39	3.71 ± 1.85	3.61 ± 1.21	Group	18.494	0.001	0.105	T1&T3	0.796	0.001			
					Time* Group	13.158	0.001	0.077	T2&T3	0.107	0.001			
Stay in the shade	Control	3.00 ± 1.67	2.65 ± 1.52	3.18 ± 1.39	Time	1.627	0.202	0.010	T1&T2	0.707	0.001	*P* = 0.765	*P* = 0.001	*P* = 0.257
	Intervention	3.07 ± 1.42	3.69 ± 1.20	3.43 ± 1.29	Group	9.997	0.002	0.060	T1&T3	1.000	0.251			
					Time* Group	5.686	0.006	0.035	T2&T3	0.004	0.339			
Wear a hat	Control	3.37 ± 1.06	3.67 ± 0.95	3.68 ± 1.06	Time	16.375	0.001	0.094	T1&T2	0.188	0.001	*P* = 0.761	*P* = 0.026	0.055
	Intervention	3.32 ± 1.35	4.00 ± 0.89	3.98 ± 0.91	Group	1.275	0.121	0.015	T1&T3	0.245	0.001			
					Time* Group	2.459	0.10	0.015	T2&T3	1.000	1.000			
Wear long-sleeved clothing	Control	4.13 ± 1.34	3.72 ± 1.21	3.84 ± 1.25	Time	1.250	0.283	0.008	T1&T2	0.109	1.000	*P* = 0.299	*P* = 0.164	*P* = 0.028
	Intervention	3.91 ± 1.38	4.01 ± 1.40	4.25 ± 1.09	Group	1.275	0.216	0.008	T1&T3	0.408	0.269			
					Time* Group	3.514	0.042	0.022	T2&T3	0.976	0.154			
Wear Sunglasses	Control	3.49 ± 1.11	3.48 ± 1.23	3.34 ± 1.20	Time	6.215	0.004	0.059	T1&T2	1.000	0.001	*P* = 0.328	*P* = 0.001	*P* = 0.001
	Intervention	3.16 ± 1.38	3.93 ± 1.15	3.87 ± 1.20	Group	2.358	0.127	0.015	T1&T3	1.000	0.001			
					Time* Group	9.113	0.001	0.077	T2&T3	0.502	1.000			
Use Sunscreen	Control	2.96 ± 1.25	3.07 ± 1.45	3.30 ± 1.37	Time	3.673	0.033	0.023	T1&T2	1.000	1.000	*P* = 0.134	*P* = 0.239	*P* = 0.193
	Intervention	3.27 ± 1.34	3.33 ± 1.29	3.58 ± 1.30	Group	3.262	0.073	0.020	T1&T3	0.257	0.287			
					Time* Group	0.023	0.965	0.000	T2&T3	0.232	0.273			
Use Sunscreen again after washing	Control	2.11 ± 1.43	2.13 ± 1.38	1.96 ± 1.21	Time	4.540	0.013	0.045	T1&T2	1.000	0006	*P* = 0.328	*P* = 0.001	*P* = 0.001
	Intervention	2.32 ± 1.23	2.86 ± 1.35	3.18 ± 1.24	Group	23.143	0.001	0.128	T1&T3	1.000	0.001			
					Time* Group	8.260	0.001	0.088	T2&T3	0.563	0.185			

Before the intervention (Time 1), there was no significant difference in the behavior of staying in the shade between the two groups (*P* = 0.765). While a significant intervention effect was observed (*P* = 0.002), there was no significant effect of time (*P* = 0.202) and interaction effect between group and time (*P* = 0.006) (Table 3).

The sun protection behavior significantly differed between the intervention and control groups immediately after the intervention (*P* = 0.001). However, after the 3-month follow-up, there was no significant difference between the intervention and control groups (*P* = 0.257). In the intervention group, a significant difference was observed from Time 1 to Time 2 (*P* = 0.001), but no significant difference was observed from Time 1 to 3 (*P* = 0.251) and from Time 2 to 3 (*P* = 0.339). In the control group, there was no significant difference observed from Time 1 to Time 2 (*P* = 0.707) and from Time 1 to 3 (*P* = 1.000), but a significant difference was observed from Time 2 to 3 (*P* = 0.004) (Table 3).

Before the intervention (Time 1), there was no significant difference in the behavior of wearing a hat between the two groups (*P* = 0.761). A significant effect of time was observed (*P* = 0.001), but there was no significant intervention effect (*P* = 0.121) and no interaction effect between group and time (*P* = 0.100). The sun protection behavior significantly differed between the intervention and control groups immediately after the intervention (*P* = 0.001) and after the 3-month follow-up (*P* = 0.055). In the intervention group, a significant difference was observed from Time 1 to 2 and also from Time 1 to 3 (*P* = 0.001), but there was no significant difference from Time 2 to 3 (*P* = 1.000). In the control group, no significant difference was observed over time (Table 3).

Before the intervention (Time 1), there was no significant difference in the behavior of wearing long-sleeved clothing between the two groups (*P* = 0.299). Although there was a significant interaction effect between group and time (*P* = 0.042), there was no significant intervention effect (*P* = 0.216) and no significant effect of time (*P* = 0.283). The sun protection behavior was not significantly different between the intervention and control groups immediately after the intervention (*P* = 0.164). However, a significant difference was observed between the two groups after the 3-month follow-up (*P* = 0.028). In both the intervention and control groups, no significant difference was observed over time (Table 3).

Before the intervention (Time 1), there was no significant difference in the behavior of wearing sunglasses between the two groups (*P* = 0.328). While there was no significant intervention effect (*P* = 0.127), there was a significant effect of time (*P* = 0.004) and a significant interaction effect between group and time (*P* = 0.001). The sun protection behavior was significantly different between the intervention and control groups immediately after the intervention (*P* = 0.001) and after the 3-month follow-up (*P* = 0.001). In the intervention group, a significant difference was observed from Time 1 to 2 (*P* = 0.001) and from Time 1 to 3 (*P* = 0.001). However, no significant difference was observed from Time 2 to 3 (*P* = 1.000). In the control group, no significant difference was observed over time (Table 3).

Before the intervention (Time 1), there was no significant difference in the behavior of using sunscreen between the two groups (*P* = 0.134). While there was a significant effect of time (*P* = 0.033), there was no significant intervention effect (*P* = 0.073), and no significant interaction effect between group and time (*P* = 0.965). The sun protection behavior was not significantly different between the intervention and control groups immediately after the intervention (*P* = 0.239) and after the 3-month follow-up (*P* = 0.193). In both the intervention and control groups, no significant difference was observed over time (Table 3).

**Fig. 3:**
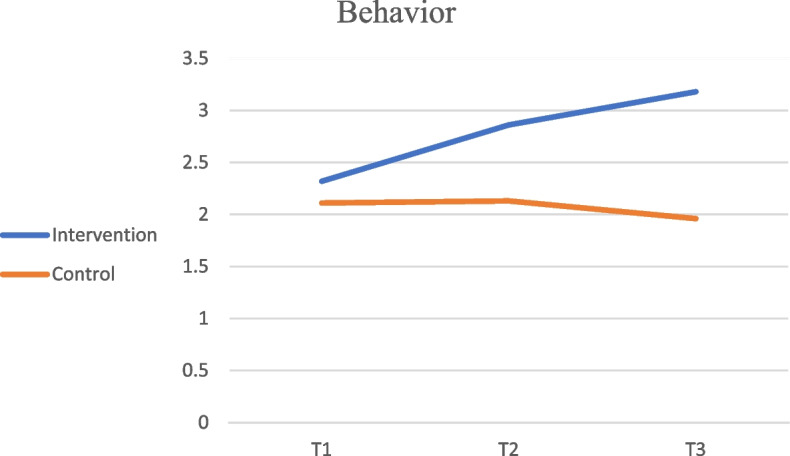
Sunscreen Reapplication Behavior After Washing Hands and Face Over Time in Intervention and Control Groups

Before the intervention (Time 1), there was no significant difference in the behavior of using sunscreen again after washing hands and face between the two groups (*P* = 0.328). However, there was a significant intervention effect (*P* = 0.001), time effect (*P* = 0.001), and interaction effect between group and time (*P* = 0.001). The sun protection behavior was significantly different between the intervention and control groups immediately after the intervention (*P* = 0.001) and after the 3-month follow-up (*P* = 0.001). In the intervention group, a significant increase was observed from Time 1 to 2 (*P* = 0.006), and while there was no significant difference between Time 2 to 3 (*P* = 0.185), there was a significant difference between Time 1 and 3 (*P* = 0.001). In the control group, no significant difference was observed over time (see Fig. 3).

## Discussion

In addressing the challenges of fostering adequate sun protection behavior among adolescents, innovative approaches are crucial [21–23]. This study explored the efficacy of a Facial-Aging web app in promoting sun protection behavior among primary school students in Iran.

In the intervention group, significant improvements were noted in key behaviors, particularly in using sunscreen again after washing hands and face, as well as using sunscreen in the last month. Sunscreen serves as a crucial second line of defense, especially for uncovered areas like the face and hands. This finding aligns with research conducted by S. Koch et al. (2017) among adolescents and women in Australia and Masoudi et al. (2014) among primary school students in Iran, emphasizing the prominence of sunscreen use as an initial sun protection measure (2014). on primary school students in Iran, showed that using sunscreen was the first sun protection behavior, which is in line with our study [24, 25]. It's noteworthy that while our study supports these findings, other studies have highlighted staying in the shade as the most commonly reported sun protection behavior [26].

Notable distinctions between the two groups were observed in four key subscales of Sun Protection Behavior: using sunscreen last month, staying in the shade, and using sunscreen again after washing hands and face. This aligns with earlier research indicating significant group disparities in sun protection behaviors, particularly in sunscreen use and hat usage [27].

Encouraging individuals, especially children, to adopt the "stay in the shade" behavior represents a simple and cost-effective means of photoprotection, particularly in school playgrounds. This consideration should be integrated into the initial design stages of new buildings, parks, and other play areas to enhance their photoprotection value for occupants [28]. A recent trial conducted in parks in Denver (USA) and Melbourne (Australia) underscored the positive impact of providing shade structures. The study revealed that people were more inclined to use passive recreation areas, such as those for socializing or watching sports when shade was available. The findings suggested that public investment in shade provision could be a worthwhile strategy for reducing the risk of skin cancer [29].

Upon closer examination of the observed pattern in sun protection behavior changes (including the use of sunscreen last month, hats, sunglasses, and staying in the shade) over time, a notable increase was evident in the intervention group from T1 to T2 compared to the control group (supporting Hypothesis 1). Moreover, significant increases were observed from T1 to T3 in the intervention group (supporting Hypothesis 2) for various sun-protective behaviors, such as using sunscreen last month, wearing hats and sunglasses, and reapplying sunscreen after washing hands and face. In contrast, participants in the control group either exhibited minimal change or displayed a decreasing trend. This trend aligns with findings from previous studies, suggesting that intervention effects tend to accelerate between 6 and 12 months, followed by a stabilization phase between 12 and 24 months. Such dynamics reinforce the effectiveness and sustainability of the implemented intervention measures [20].

The optimal approach to safeguarding both the skin and eyes involves combining protective measures, such as wearing hats and sunglasses. Hats play a crucial role in shielding the scalp and casting shade over various facial regions, offering substantial protection. The effectiveness varies based on the hat type and the sun's angle. Notably, a wide-brimmed hat stands out as the most effective, providing extensive coverage and excellent protection for the forehead, nose, ears, cheeks, and neck [30]. Furthermore, sunglasses contribute significantly to shielding against UV radiation, offering excellent eye protection [31]. The combined use of hats and sunglasses thus forms a comprehensive strategy for ensuring robust protection for both the skin and eyes.

Exposure to sunlight constitutes a recognized or potential risk factor for various eye conditions that can lead to moderate or severe visual impairment. Examples include cataracts and age-related macular degeneration [32]. Despite the considerable impact of these eye diseases, there is a notable lack of awareness regarding the importance of sun protection for the eyes. For instance, in a cross-sectional study involving university students in northern China (n = 386), more than 90% were informed about the effects of UV radiation on skin-related issues like sunburn and skin cancer. However, only 28% were aware of the heightened risk of cataracts, and a mere 3% recognized the risk of pterygium. Additionally, protective measures for the eyes during sun exposure were infrequent [33].

In the intervention group, there was no significant difference in sun protection behaviors, including staying in the shade and wearing long-sleeved clothes, from Time 1 to 3 and from Time 2 to 3. This lack of significant change could potentially be attributed to regional and cultural differences. The study area is situated in one of the southern regions of Iran, characterized by minimal vegetation and heightened drought conditions [34]. Consequently, the scarcity of greenery and tall trees in this region makes it challenging to find adequate shade.

Concerning the behavior of wearing long-sleeved clothing, students scored more than 80%. This high score can be attributed to the traditional clothing in the region, particularly women's clothing, which incorporates various components aligned with the climatic, cultural, and historical conditions of the area. The traditional attire provides ample coverage [35]. Hence, in this study, the use of sun protection clothing may be more linked to the cultural expectations of the region's attire rather than a deliberate practice of sun protection.

Geographic location can impact socioeconomic status, cultural values, and social norms. Our research identified regional variations in sun-protective behaviors, such as staying in the shade and wearing long-sleeved clothing. This contrasts with previous studies that indicated geographic region might not play a decisive role in sun protection practices [36].

Our study has several limitations. Firstly, our survey data collection coincided with the COVID-19 pandemic, impacting school closures and limiting sunlight exposure. This situation warrants consideration for potential biases. Secondly, the absence of parental involvement is noteworthy, given their crucial role in purchasing protective measures. Future studies are advised to include parental perspectives and replicate the study with younger children. Thirdly, relying on self-reported questionnaire data introduces the possibility of social desirability bias. Acknowledging this limitation is essential for interpreting participant responses accurately. Additionally, the study faced constraints related to opt-in participation, hinging on access to smartphones and internet connectivity. Addressing these challenges in future intervention studies is recommended for a more inclusive approach.

## Conclusions

The notable improvement in sun protection behaviors observed among students in the intervention group, as compared to the control group, underscores the efficacy of the Facial-Aging web app in promoting sun-safe practices. This study not only assessed the impact of innovative appearance-based interventions but also demonstrated their scalability among students. Integrating such interventions with other strategies could form a comprehensive approach to enhance students' sun protection behaviors. To ensure the durability of these positive behavioral changes, it is recommended that future research explores the effectiveness of implementing booster sessions.

### Supplementary Information

**Supplementary Material 1. **

## Data Availability

The datasets generated and/or analyzed in the study are available from the corresponding author upon reasonable request.
