# Supplementary material for: Efficacy of a facial-aging web app on sun protection behaviors among primary school students in Iran: a randomized controlled trial
Source: BMC Public Health. 2024 Mar 7;24:737. doi: 10.1186/s12889-024-18241-2 (PMC10921649; doi:10.1186/s12889-024-18241-2)
Supplement: Supplementary file 1 — Supplementary Material 1. [file 12889_2024_18241_MOESM1_ESM.docx]

Appendix1

Health messages grounded in the Protection Motivation Theory

| Variables | Messages |
| --- | --- |
| Perceived susceptibility | SMS number 1:  Dear children,  Did you know that skin cancer is the most prevalent type of cancer in many Iranian cities, including Sistan and Baluchistan?  SMS number 2:  Dear children,  Remember, exposure to sunlight is a contributing factor to skin cancer and eye damage in adulthood. Everyone is at risk, but individuals with light skin and colored eyes face a higher risk of skin cancer and eye damage in adulthood. Stay protected! |
| Perceived severity | SMS number 1  SMS number 1:  Dear children, two important points:  1- Skin cancer is a dangerous disease  2- Eye damage caused by sunlight, including cataracts, is a serious health problem.  SMS number 2:  Dear children, keep in mind:  1) If skin cancer is not diagnosed in time, it can lead to a person's death.  2) Although there is no definitive treatment for skin cancer, it is easily preventable. |
| Fear | SMS number 1:  Feeling worried about skin cancer is normal. Stay with us, and in our next SMS, we'll share tips on how you can overcome this fear.  SMS number 2:  The fear of skin cancer might make you uncomfortable, but by following our next SMS, you'll discover that this fear can be eliminated by taking certain actions. |
| Self-efficacy, response cost, and perceived response efficacy | SMS number 1:  "Health is your greatest wealth. Discover how to protect yourself from skin cancer. Please follow our SMS."  SMS number 2:  "Dear student, don't be lazy. While using sunscreen may take time, it's a crucial step in preventing skin cancer."  SMS number 3:  "Dear student, although standard sunglasses can be expensive, you can save money by asking your parents or older siblings to get them for you."  SMS number 4:  "If buying sunscreen and sunglasses is challenging, take care of yourself by wearing clothes and staying in the shade."  SMS number 5:  "Dear student, wearing a hat may seem inconvenient, but if your health is a priority, consider making the serious decision to wear a hat regularly."  SMS number 6:  "Can you sit in a car without a seatbelt? Sunscreen is your sunbelt. Make it a habit to protect yourself from the sun."  SMS number 7:  "Buying foreign sunscreens can be costly. You can opt for high-quality Iranian sunscreens at a much lower price."  SMS number 8:  "Don't worry about classmates making fun of you for wearing sunglasses. Your eye protection is more important."  SMS number 9:  "Though long-sleeved clothes may make you feel hot, they reduce your skin's exposure to the sun."  SMS number 10:  "I know playing with a hat may not be easy, but you can try for the sake of your health."  SMS number 11:  "If you forget sunscreen at home, keep a small bottle in your bag for on-the-go protection."  SMS number 12:  "My dear friends, using sunglasses is not just for looks. Educate others about the importance, and encourage them to protect their eyes too." |
| Reward | SMS number 1:  "Protecting yourself from the sun not only prevents skin cancer but also other diseases, including cataracts."  SMS number 2:  "Playing without a hat may be easy, but wearing one can protect your eyes and skin from the sun."  SMS number 3:  "While you might feel more comfortable without sunglasses and a brimmed hat, wearing them ensures protection for your eyes and skin from the sun." |
| Protection motivation | SMS number 1:  "Now is the time to decide! What is your decision? We've been with you for a week, sincerely reminding you. It's your turn to decide."  SMS number 2:  "Never say it's too late; make a decision now. I intend to stay indoors during peak sunlight hours and postpone outdoor activities."  SMS number 3:  "Never say it's too late; make a decision now. If sunscreen or sunglasses are expensive, at least choose to be in the shade when commuting or engaging in activities."  SMS number 4:  "Never say it's too late; make a decision now. Always carry sunscreen with you and use it regularly every day."  SMS number 5:  "Sunscreen is not just for summer; it should be used in all seasons—autumn, spring, and winter."  SMS number 6:  "Know that health is your inalienable right. As God's creation, you deserve to live a life full of health and happiness." |
